# Causal Attribution and Coping Maxims Differences between Immigrants and Non-Immigrants Suffering from Back Pain in Switzerland

**DOI:** 10.1371/journal.pone.0161758

**Published:** 2016-09-01

**Authors:** Sarah Mantwill, Peter J. Schulz

**Affiliations:** Institute of Communication & Health, University of Lugano, Lugano, Switzerland; Leibniz Institute for Prvention Research and Epidemiology BIPS, GERMANY

## Abstract

**Objectives:**

This study aimed at investigating the relationship between causal attributions and coping maxims in people suffering from back pain. Further, it aimed at identifying in how far causal attributions and related coping maxims would defer between immigrants and non-immigrants in Switzerland.

**Methods:**

Data for this study came from a larger survey study that was conducted among immigrant populations in the German- and Italian-speaking part of Switzerland. Included in the analyses were native Swiss participants, as well as Albanian- and Serbian-speaking immigrants, who had indicated to have suffered from back pain within the last 12 months prior to the study. Data was analyzed for overall 495 participants. Items for causal attributions and coping maxims were subject to factor analyses. Cultural differences were assessed with ANOVA and regression analyses. Interaction terms were included to investigate whether the relationship between causal attributions and coping maxims would differ with cultural affiliation.

**Results:**

For both immigrant groups the physician’s influence on the course of their back pain was more important than for Swiss participants (*p* <.05). With regard to coping, both immigrant groups were more likely to agree with maxims that were related to the improvement of the back pain, as well as the acceptance of the current situation (*p* <.05). The only consistent interaction effect that was found indicated that being Albanian-speaking negatively moderated the relationship between physical activity as an attributed cause of back pain and all three identified coping maxims.

**Conclusion:**

The study shows that differences in causal attribution and coping maxims between immigrants and non-immigrants exist. Further, the results support the assumption of an association between causal attribution and coping maxims. However cultural affiliation did not considerably moderate this relationship.

## Introduction

Research has shown that causal attributions of illnesses can have differential effects on health behaviors [1] and health outcomes [2, 3], as well as on adjustment and coping behaviors [4–7]. As postulated by Leventhal`s common sense model of self-regulation of health and illness [8], individuals create explanations of their illnesses (i.e. abstract illness representations) that subsequently guide decisions and behaviors related to the illness. The selection of a health behavior, whether preventive or acute, will depend on the fit between the individual`s illness representation, the procedure itself and an action plan [9–11]. Thus, coping decisions will be reflective of illness representations, which in turn might be reflective of past illness experiences, available information, and other environmental factors [12]. Illness representations can be divided into five attributional dimensions, among those the identity of the illness (how people label their illness), the duration of the illness (timeline) and those beliefs that this article is concerned with, namely the causes of an illness [13–15]. Work on illness representations has identified a number of causal factors that people attribute their illnesses to, including biological (e.g. germs and viruses), emotional (e.g. stress and depression), environmental (e.g. pollution), and psychological causes (e.g. personality). However, classifications of causal attributions still largely vary. [16]

Coping strategies are often classified into active and passive. In chronic pain patients, active coping strategies have been linked to positive adjustment, such as reduced depression levels or better psychological adjustment. On the other hand, passive coping strategies have been associated with negative outcomes, such as increased depression levels and pain severity. [17] The evidence on how attributions are related to coping behavior is still not coherent [6]. Roesch and colleagues [6] found in their meta-analysis that attributing an illness to internal or controllable causes for example was more likely to lead to approach coping, which refers to a coping style that is actively directed at the pain and related feelings. It may include active information seeking, problem solving or search for social support [18]. More specifically, with regard to pain coping, one study identified that causes of back pain that were perceived as being influencable by participants were related to preferences for more active coping styles in people suffering from back pain [19]. Hoffman and colleagues [20] found in their qualitative study, that low back pain patients believed that the identification of the cause of the back pain was crucial for effective treatment. Also, it has been observed that in chronic pain patients, the belief in organic causes and consequences of pain was related to the belief that others (e.g healthcare practitioners) control the pain [21]. Further, a study in arthritis patients found that causal attribution significantly mediated the relationship between perceived control, respectively self-efficacy, and depression or disability [22]. On the other hand, Kraimaat and colleagues [23] found that causal attribution did not considerably contribute to pain-coping behavior in persons suffering from chronic headache.

The literature shows that racial and ethnic minorities, as compared to the racial/ethnic majority, cope differently with pain-related conditions [24–26]. In the US for example African-Americans have been found to be more prone to use passive coping strategies, including praying and hoping [27]. A recent meta-analysis on pain coping partially confirmed these results and ascertained that overall African-Americans, as compared to White Americans, used pain coping strategies more frequently. The largest differences were found for praying and catastrophizing, suggesting that African-Americans use more often those strategies that are related to poorer pain outcomes. [28] Similar patterns have also been identified in Europe, where immigrants have been found to rely more frequently on passive coping strategies, such as catastrophizing [29, 30].

According to data from the Swiss Federal Statistical Office, in 2012 about 40% of the population in Switzerland indicated to have suffered within the last 4 weeks from back pain (including low back pain) [31]. Further, based on data from 2005 it was estimated that low back pain alone created direct medical costs of 6.1% of the total Swiss healthcare expenditure [32]. In particular among immigrants and asylum-seekers in Switzerland back pain is one of the most frequently cited health complaints [33, 34]. About 35% of the Swiss population has an immigration background (including first and second generation immigrants) [35] and the largest two groups of first generation immigrants that are not native speakers of one of the official Swiss languages are, after Portuguese immigrants (ca. 271`000), Albanian- (of whom the majority comes from Kosovo, ca. 100`000) and Serbian-speakers from Serbia (ca. 78`000) and Bosnia and Herzegovina (ca. 33`000) [36]. Data shows that especially immigrants who do not speak one of the official Swiss languages are more likely to be negatively affected by health disparities [37]. Differences can be seen for example for preventive healthcare usage [38–40] and studies have shown that immigrants, compared to the Swiss native population, are more likely to be overweight and less likely to be physically active [37, 38, 41, 42]. One study from Switzerland found that among chronic pain patients, immigrants suffered from higher pain severity and worse psychological functioning. They were also more likely to engage in passive coping behavior, in particular catastrophizing. Further, the study showed that also after an 8-week intervention changes in immigrants were comparably small. [29]

### Objective

Based on previous research conducted by Schulz and colleagues [19] (see above), the objective of this study was to investigate the relationship between causal attributions and coping maxims among immigrants with an Albanian- and Serbian-speaking background in the German- and Italian-speaking parts of Switzerland and to compare them to the Swiss native population.

Schulz and colleagues [19] had investigated micro-cultural differences in causal attribution and coping maxims among Swiss natives suffering from back pain across the three language regions in Switzerland. Amongst others, the study found that participants from the German-speaking part preferred active coping maxims as compared to French-speaking participants who preferred passive coping maxims. Further, job stress was more likely to influence coping behavior related to improvement of the current condition when the person was Italian-speaking. [19]

The assumption for the study at hand was that cultural affiliation, as defined by immigrant group, would amplify the differences between immigrants and non-immigrants in causal attribution and coping maxims among persons suffering from back pain. In addition, the study hypothesized that casual attributions would be independently associated with coping maxims across the different groups and that cultural affiliation would explain some of the potential differences.

## Methods

Data for the study at hand was collected during summer/fall 2013 in the context of a larger project, which aimed at investigating different aspects of influence factors, coping behavior and the interaction with the healthcare system among gainfully employed immigrants and non-immigrants in the German- and Italian-speaking part of Switzerland suffering from back pain.

Data was collected via self-administered paper and pencil, as well as online questionnaires using snowball sampling. Recruitment of participants took place via trained recruiters who were native speakers of the respective languages of each group. Further, participants were also recruited via cultural organizations, as well as community outreach by identifying local community members who were able to promote the study [43, 44]. Participants could fill in the questionnaire in the language of their choice, namely German, Italian, Albanian, or Serbian. In order to be eligible to participate in the study, participants had to have suffered from back pain (including lumbago and cervical pain), which was assessed with a self-reported question at the beginning of the survey *(“In the last 12 months*, *have you suffered from back pain (including lumbago and cervical pain)*?*”)*. Participants had to have been gainfully employed. Further, only first- or second-generation immigrants were eligible to participate. As a thank you participants had the option to participate in a prize draw.

### Measures

Even though the complete survey contained a number of different scales, the study at hand focused on only two measures that investigated (1.) causal attributions of back pain and (2.) coping maxims directed at back pain. The measures, as well as the relevant analyses, were based on the study conducted by Schulz et al. [19]. The German and Italian versions of the scales were taken from the original questionnaires, whereas the Albanian and Serbian versions were translated from English into the respective languages using appropriate translation techniques (including forward- and back-translations). Discrepancies between translations were resolved by consensus finding among the translators and project coordinators. Questionnaires were subsequently pretested and, if necessary, adaptations were made.

#### Causal attributions

Schulz et al. [19] differentiate between original causes of the back pain and influence factors on the course of the back pain (causal attributions). In comparison to the original causes, influence factors are considered to be more closely linked to coping behavior, as they relate to the potential recovery from the back pain. After consultation with the authors of the original scale, it was decided to drop three of the original items, including two items that had been excluded in the original exploratory factor analysis of the scale [19]. This left a scale of overall 17 items. Causal attributions were assessed with two different questions, asking about long-term and short-term influence factors [19]. Eight items inquired about the long-term influence factors (*“What does the improvement*, *or at least the not worsening*, *of your back pain in the long-term depend on*?*”*) and nine items about the short-term influence factors (“*Along with the long-term influences on back pain*, *there may be circumstances which determine the onset or the worsening of your back pain for a short term*.*”*). Answer options for long-term influence factors included for example: *“Being regular with my physician visits”* or *“Regular exercise”*. Answers were scored on a Likert-type scale ranging from 0 = “Has no influence” to 6 = “Has a very strong influence”.

#### Coping maxims

Coping maxims were defined in terms of simple behavioral rules or intentions towards the back pain [19]. Also here the original scale was used [19] and all 12 items were included in the analysis. Participants were asked to respond to twelve statements that asked them in how far each individual statement would apply to them (Ex. *“I should avoid movements that could worsen my pain*.*”)*. Answers were assessed with a Likert-type scale, ranging from 0 = “Not at all” to 6 = “Completely”.

### Data Analysis

Confirmatory, respectively exploratory, factor analyses were run in order to determine the factor structure of the two scales measuring causal attributions and coping maxims. For the exploratory factor analysis the minimum loading of an item was set at .30.

To determine the most appropriate factor model in the CFA, several fit indices were assessed, including the root mean square error approximation (RMSEA), goodness of fit index (GFI), adjusted goodness of fit index (AGFI) and comparative fit index (CFI). Further, in case of problematic items, the standardized residual covariance matrix was investigated.

Factor scores of the latent variables were calculated. Subsequently, ANOVAs were run to investigate differences between the different groups for causal attribution and coping maxims. Next, multiple stepwise regressions were conducted in order to investigate the link between causal attribution and coping maxims. Finally, interaction terms were included in order to test for the possible interaction between immigrant group and causal attribution. To control for potential micro-cultural differences between Swiss participants [19, 45, 46], additional analyses were run dividing Swiss participants into German- and Italian-speaking participants.

Data was initially checked for missing values and was found to be missing completely at random (MCAR), *p* = .174. In order to run the CFA, missing values were imputed using the expectation maximization technique. Analyses were computed with SPSS 23.0 and AMOS 23.0.

### Ethics Statement

The research, including implied consent, was approved by the review board of the Canton Ticino in Switzerland (Bellinzona, Switzerland). In two cases participants under 18 years of age were included, when questionnaires were conducted in family settings. Participation was verbally approved by guardians. In all cases implied consent was obtained by (1.) providing an informational letter and (2.) the completion and submission of the survey (online or via postal mail).

## Results

Data for 495 participants was analyzed in this study. 247 participants were from the Italian-speaking part and 247 from the German-speaking part of Switzerland. 27.9% (n = 138) of the participants had an Albanian-speaking background, 39.6% (n = 196) had a Serbian-speaking background (hereafter referred to as Albanian-speaking and Serbian-speaking) and 32.5% were Swiss participants (Swiss-Italian: n = 91; Swiss-German: n = 70). Slightly more men (n = 254) than women (n = 223) participated in the survey. (Table 1) Most participants were in the age group 21–40 (n = 256). The overall sample showed a relatively high educational level; most people had either a high school diploma or had finished vocational training (n = 176), followed by participants who had attended institutions of higher education (n = 152).

**Table 1:** Socio-demographics of the overall sample.

Demographic	Language Groups				
	Albanian-speaking (%)	Serbian-speaking (%)	Swiss-German (%)	Swiss-Italian (%)	*p*
**Age**					
16–20	3 (2.2)	14 (7.3)	1 (1.4)	5 (5.8)	
21–40	79 (59.0)	119 (62.0)	29 (42.0)	29 (33.7)	
41–60	51 (38.1)	52 (27.1)	38 (55.1)	49 (57.0)	
61 and above	1 (0.7)	7 (3.6)	1 (1.4)	3 (3.5)	*<*.*01*
**Gender**					
Female	39 (29.5)	97 (50.8)	34 (49.3)	53 (62.4)	
Male	93 (70.5%)	94 (49.2)	35 (50.7)	32 (37.6)	*<*.*01*
**Education**					
No degree/elementary school	32 (24.4)	8 (4.2)	0 (0.0)	3 (3.5)	
Secondary school	41 (31.3)	53 (27.7)	4 (5.9)	6 (7.1)	
High school/vocational training	40 (30.5)	51 (26.7)	30 (44.1)	55 (64.7)	
Higher educational degree	18 (13.7)	79 (41.4)	34 (50.0)	21 (24.7)	*<*.*01*
**Region of Residence**					
German-speaking part	82 (59.4)	96 (49.0)	68 (97.1)	1 (1.1)	
Italian-speaking part	56 (40.6)	100 (51.0)	2 (2.9)	90 (97.8)	*<*.*01*

324 participants indicated to have suffered regularly from back pain for more than 3 months. 189 participants had already received a diagnosis with regard to their back pain.

### Factor Analysis

#### Causal attributions

Confirmatory factor analysis (CFA) and explanatory factor analysis (EFA) were used to determine the factor structure. A first CFA did not reveal the same five-factor structure as described by Schulz and colleagues (2013), and model fitting did not correct for the problem. Subsequently an EFA with principal-axis factoring and promax oblique rotation was run, since factors were assumed to be correlated with each other [47]. A five-factor solution was revealed: (1.) Burden and Fatigue, (2.) Physician`s Influence, (3.) Emotions and Mood, (4.) Climate, and (5.) Physical Activity (when appropriate, factors names were kept as close as possible to the original scale from Schulz et al. [19]). (Table 2) Two items were excluded from further analysis as they did not sufficiently load on one of the factors (<.30). Next a CFA was conducted and it was decided to drop two additional variables due to their low loadings. The final model showed a good fit (RMSEA: .057; PCLOSE: .163; GFI: .958; AGFI .930; CFI: .957) [48].

**Table 2:** Confirmatory factor analysis of causal attributions.

	Factors				
	1	2	3	4	5
	Burden and Fatigue	Physician`s Influence	Emotions and Mood	Climate	Physical Activity
The workload (long-term)	.80				
Specific physical stress at work (short-term)	.76				
My fatigue (long-term)	.64				
Being regular with my physician visits (long-term)		.77			
Following my doctor’s orders/medical advice (long-term)		.76			
The physicians’ competence (long-term)		.70			
My own mood (short term)			.70		
Specific psychological stress at work (short-term)			.67		
Fate, i.e. whether I have good or bad luck (long-term)			.56		
The weather (e.g. cold weather, humidity) (short-term)				.89	
Draught/Current of air (short-term)				.73	
Physical exercise (short -term)					.73
Regular exercise (long-term)					.58

Long-term influence factors: *“What does the improvement*, *or at least the not worsening*, *of your back pain in the long-term depend on*? *Please answer this question using a scale from 0 to 6 where 0 indicates "Has no influence" and 6 "Has a very strong influence*”. *My back pain is influenced in the long term by the following factors*:*…”;* Short-term influence factors: “*Along with the long-term influences on back pain*, *there may be circumstances which determine the onset or the worsening of your back pain for a short term*. *Please indicate to what extent the following circumstance do influence your back pain using a scale from 0 to 6 where 0 indicates “Has no influence” and 6 “Has a very strong influence”*.*”*

#### Coping maxims

Similar to the previous analysis, the initial CFA did not reveal a similar factor structure; hence an EFA was run (principal-axis factoring, promax oblique rotation). One item was dropped and a three-factor structure seemed to be the most appropriate one. After elimination of an additional item the model showed a good fit (RMSEA: .058; PCLOSE: .178; GFI: .970; AGFI: .948; CFI: .950) [48]. Based on the previous study [19] the three factors were named the following: (1.) Aspiration to Improvements; (2.) Acquiescence in the Condition; (3.) Acceptance of Blame. (Table 3)

**Table 3:** Confirmatory factor analysis of coping maxims.

	Factors		
	1	2	3
	Aspiration to Improvement	Acquiescence in the Condition	Acceptance of Blame
I will be able to fully enjoy life again only once I’m free of back pain.	.75		
My goal for the future is to be able to go back to doing everything that I used to do before the onset of my condition.	.69		
I should avoid movements that could worsen my pain.	.65		
The improvement of my back comes first, sometimes work must come second.	.58		
Even if others look at me in a strange way, I take measures to protect and support my back, for e.g. I sit on an exercise ball (psychomotor ball) instead of a chair.	.46		
Since people are not able to see my pain, they probably think that I am only pretending to have back pain.		.66	
When I am in pain, it makes me feel good when I can talk to other people about it.		.63	
I am more or less used to my back pain; it has become part of my life.		.41	
I feel a bit guilty because I am not doing enough for my back.			.93
It is my own responsibility if my condition does not improve.			.47

“To what extent do the following statements apply to you? Please answer this question using a scale from 0 to 6 where 0 indicates “Not at all” and 6 “Completely”.”

### Differences between Immigrants and Non-Immigrants

Albanian- and Serbian-speakers were significantly more likely than Swiss participants to attribute their back pain to the “Physician`s Influence”. Compared to the Swiss population, Albanian-speakers attributed their back pain more frequently to “Burden and Fatigue” and the “Climate”. On the other hand, Serbian-speaking participants, compared to Swiss participants, were significantly more likely to attribute their back pain to their emotional state. (Table 4) Interestingly, when Swiss participants were separated into the two language groups, it was found that Swiss-Italian participants were the least likely to attribute their back pain to their emotional state.

**Table 4:** Mean factor scores of causal attribution and coping maxims.

	Albanian-speaking (n = 138)	Serbian-speaking (n = 196)	Swiss (n = 161)	F	*p*
**Causal Attribution Factors**					
Burden and Fatigue	3.81	3.28^a^	3.14^a^	9.671	<.001
Physician`s Influence	3.16	2.72	2.02	31.750	<.001
Emotions and Mood	2.24^ab^	2.32^b^	1.97^a^	4.360	<.05
Climate	3.77	3.27^a^	2.92^a^	9.810	<.001
Physical Activity	2.05^ab^	1.98^ac^	1.90^bc^	1.082	Ns
**Coping Maxims**					
Aspiration to Improvements	2.64^a^	2.50^a^	1.98	20.366	<.001
Acquiescence in the Condition	2.84^a^	2.78^a^	2.01	30.883	<.001
Acceptance of Blame	1.53^ab^	1.70^a^	1.30^b^	10.063	<.001

Causal attribution: df = 2, 492; Coping maxims: df = 2,429; Same superscript letters indicate non-significant pairwise comparisons, as assessed with appropriate post-hoc tests (Gabriel or Games-Howell test)

With regard to coping maxims, participants with an immigration background were significantly more likely to agree with the coping maxims “Aspiration to Improvement” and “Acquiescence in the Condition” than Swiss participants. Further, Serbian-speakers were the most likely to agree with “Acceptance of Blame”. (Table 4) When divided into the two language groups, Swiss-Italians were the least likely to agree with “Acceptance of Blame”.

### Regression Analysis

Multiple stepwise regression analyses were computed, to investigate which variable(s) would best predict the three different coping maxims: (1.) Aspiration to Improvement; (2.) Acquiescence in the Condition; (3.) Acceptance of Blame. In the first step the five identified causal attributions, as well as cultural affiliation (as defined by the two immigrant groups) were inserted. In a second step the interaction terms between cultural affiliation and causal attributions were added to the model. The final regression models included only those interactions that had shown to be significant. (Table 5)

**Table 5:** Effects of causal attributions on coping maxims.

	Coping Maxims		
	Aspiration to Improvement	Acquiescence in the Condition	Acceptance of Blame
**Causal Attribution**			
Burden and Fatigue	.050	-.019	.033
Physician`s Influence	.205**	.152*	-.066
Emotions and Mood	.207**	.288***	.047
Climate	.190**	.204***	.095
Physical Activity	-.060	-.019	.256***
**Culture**			
Albanian-speaking	.148**	.204***	.096
Serbian-speaking	.149**	.237***	.216***
(Adj. R^2^)	(.318)	(.354)	(.122)
**Interactions**			
**Albanian-speaking**			
Burden and Fatigue			
Physician`s Influence			
Emotions and Mood			.140*
Climate			
Physical Activity	-.120**	-.088*	-.177**
**Serbian-speaking**			
Burden and Fatigue			
Physician`s Influence			
Emotions and Mood			
Climate			
Physical Activity			
(Adj. R^2^)	(.327)	(.359)	(.135)

***p <.001;

**p <.01;

*p <.05.

#### Outcome “aspiration to improvement”

Being Albanian- or Serbian-speaking was significantly associated with the coping maxim “Aspiration to Improvement”. Further the causal factors that were significantly associated with the outcome were “Physician`s Influence”, “Emotions and Mood” and “Climate”.

When tested for interaction effects between cultural affiliation and causal factors, the analysis found only one significant interaction effect for Albanian-speaking participants. The more Albanian-speakers ascribed their back pain to physical activity, the less did they favor “Aspiration to Improvement” as a coping maxim. (Table 5)

#### Outcome “acquiescence in the condition”

Similar to the previous findings, being Albanian- or Serbian-speaking was significantly associated with “Acquiescence in the Condition”. Also “Physician`s Influence”, “Emotions and Mood” and “Climate” were again significantly associated with the outcome. Adding interaction effects to the model showed that the same previously identified interaction effect between Albanian-speaking and “Physical Activity” emerged.

#### Outcome “acceptance of blame”

Compared to the two other outcomes, the analysis showed that none of the causal attributions, except for “Physical Activity”, was significantly associated with “Acceptance of Blame”. When adding interaction terms it was found that again “Physical Activity” and being Albanian-speaking was significantly associated with the outcome. In addition, the analysis also showed that the more Albanian-speakers ascribed their back pain to their emotional state, the more they accepted “Acceptance of Blame” as a coping maxim.

When the analysis was split into four groups (Swiss participants split into two language regions) it was found that even though no significant interaction effects were found for Italian-speakers, being Swiss-Italian was the only variable that was significantly associated with “acceptance of blame”.

## Discussion

In how far cultural differences play a role for illness representations has so far received little empirical attention [19]. Therefore the current paper aimed at investigating causal attributions and coping maxims with regard to back pain and in how far cultural affiliation, as defined by immigrant vs. non-immigrant background, would influence the relationship between these two constructs. It was hypothesized that causal attribution was independently associated with coping maxims. Overall the results of this study support the hypothesized relationship between causal attributions and coping maxims. Yet, the results also show that cultural affiliation, as a potential mediating factor, seems to play only a minor role in this relationship.

Overall, the study showed that immigrants were more likely to attribute their back pain to “Physician`s Influence”, meaning that they believed that their back pain would depend, for example, on them following their physician`s orders or on their regularity with physician visits. Part of the explanation might lie in the fact that most of the participants with an immigration background in this study came from countries that until fairly recently still had a very paternalistic vision on healthcare, thus ascribing the physician a very important role in treating illnesses [49, 50]. The study further identified that Albanian-speakers were more likely to attribute their back pain to “Burden and Fatigue” and “Climate”. Both results are not necessarily surprising, given that two thirds of Albanian-speaking participants in our sample had indicated that their current occupation would require “some” to “very vigorous” physical efforts (not shown in results). In addition, Swiss data has shown that a large part of the Albanian-speaking population in Switzerland still works mostly outside, for example in construction or the agricultural sector [51].

The official health monitoring of the immigrant population in Switzerland [38, 52] has found a tendency for people with an immigration background to develop psychological problems (depressions) with increasing age. In the literature depression and back pain have been frequently associated with each other [53, 54]. Amongst others it has been found that depression increases with pain severity [53]. However causal effects seem to work both ways [55] and only recently research has started to look into how mood might influence pain [56]. In a broader context this might explain the study`s finding that Serbian-speakers, as compared to Swiss participants, were more likely to attribute their back pain to their own emotional state. Their perception of their emotional state might perpetuate their perception of the severity of their back pain.

Even though differences between the groups with regard to causal attribution and coping maxims were found, the association between the two constructs was not necessarily explained by cultural affiliation. The only consistent relationship was found for “Physical Activity” and being Albanian-speaking. Initially no differences between the groups with regard to “Physical Activity” had been identified, however, in the regression analyses it was found that the more Albanian-speakers ascribed their back pain to “Physical Activity” the less they were likely to agree with the coping maxims. Interestingly, even though not significant, the same trend was found for Serbian-speakers.

As already mentioned, a large part of participants with an immigration background had indicated to work in positions that require physical efforts. Further, most of the Albanian- as well as Serbian-speaking participants had indicated to work in craft and skilled, personal services, as well as in operative and assembly, and elementary (unskilled) services (not shown in results). Besides the physical strains that these occupations impose, they do not necessarily follow regular working schedules, which may take its toll on voluntary physical activity, eventually negating the effect of a possibly positive influence factor on coping maxims [57–60].

Overall, it is also noteworthy that “Physical Activity” was the only causal factor that was significantly associated with “Acceptance of Blame”. This finding might be well in line with Weiner`s [61] attribution theory, which describes amongst others that the recognition of a causal factor that was initially perceived as being controllable (in this case physical activity) might lead to feelings of guilt [6]. In this regard, the effects found for Albanian-speaking participants might be further explained by the fact that even though physical activity is understood as an important causal influence factor, its potential uncontrollability due to one`s own working schedule might turn physical activity into an uncontrollable factor, eventually leading to decreased believe in effective coping strategies [6].

## Limitations

This study has several limitations. First of all, the study did not control for other socio-demographic variables, such as type of employment, and did not include measures e.g. on pain severity, other chronic conditions or depression level. Based on Schulz and colleagues`[19] study, it was decided to focus solely on the theoretical relationship on how causal attributions might contribute to coping maxims, while controlling for cultural affiliation. Further, some of the factors included in the analysis consisted of only two items, which can lead to unstable factor structures. However, these factors partly overlap with two-item factors identified by the previous study [19]. Future studies will have to generate additional items in order to create a more stable structure. An additional limitation of the study is that it did not differentiate between first and second-generation immigrants. Given the relatively small number of second-generation participants, it was deemed unfeasible to run separate analyses. Future studies should investigate the effects of possible acculturation and whether differences occur between first- and second-generation immigrants in the context of back pain. Further, the study at hand used snowball sampling in order to recruit participants, meaning that one cannot exclude the possibility that participants, especially those with an immigration background, came from relatively socially cohesive groups [62]. In addition, due to the design of the study, people who had lower levels of literacy might have been excluded from the sample. Further, the study included only gainfully employed or self-employed participants, making it thus not completely representative of the populations at large. Lastly, even though participants with an immigration background agreed more frequently with “Aspiration to Improvement” and “Acquiescence in the Condition”, these results need to be interpreted very carefully, as the study did not include, for example, a measure of pain intensity that could have explained some of the variance found.

## Conclusion

This study has important implications for clinical practice and future research. The results show that differences in causal attribution and coping maxims between immigrants and non-immigrants exist. Healthcare providers and public health specialists should be aware of potential differences in order to provide and discuss appropriate information with patients and those likely to be affected by back pain. Overall, the results of our study also seem to point to a rather emotional response towards back pain among the immigrant population. Future research should therefore look into possible mediating factors, such as social support or acculturation to explain some of the differences found for immigrants.

## Supporting Information

S1 File Dataset. Casual Attribution—Coping Maxms.sav.
(SAV)
